# PDL1Binder: Identifying programmed cell death ligand 1 binding peptides by incorporating next-generation phage display data and different peptide descriptors

**DOI:** 10.3389/fmicb.2022.928774

**Published:** 2022-07-15

**Authors:** Bifang He, Bowen Li, Xue Chen, Qianyue Zhang, Chunying Lu, Shanshan Yang, Jinjin Long, Lin Ning, Heng Chen, Jian Huang

**Affiliations:** ^1^Medical College, Guizhou University, Guiyang, China; ^2^School of Healthcare Technology, Chengdu Neusoft University, Chengdu, China; ^3^School of Life Sciences and Technology, University of Electronic Science and Technology of China, Chengdu, China

**Keywords:** PD-1/PD-L1 pathway, PD-L1 binding peptides, machine learning, next-generation phage display (NGPD) biopanning, support vector machine (SVM)

## Abstract

Monoclonal antibody drugs targeting the PD-1/PD-L1 pathway have showed efficacy in the treatment of cancer patients, however, they have many intrinsic limitations and inevitable drawbacks. Peptide inhibitors as alternatives might compensate for the drawbacks of current PD-1/PD-L1 interaction blockers. Identifying PD-L1 binding peptides by random peptide library screening is a time-consuming and labor-intensive process. Machine learning-based computational models enable rapid discovery of peptide candidates targeting the PD-1/PD-L1 pathway. In this study, we first employed next-generation phage display (NGPD) biopanning to isolate PD-L1 binding peptides. Different peptide descriptors and feature selection methods as well as diverse machine learning methods were then incorporated to implement predictive models of PD-L1 binding. Finally, we proposed PDL1Binder, an ensemble computational model for efficiently obtaining PD-L1 binding peptides. Our results suggest that predictive models of PD-L1 binding can be learned from deep sequencing data and provide a new path to discover PD-L1 binding peptides. A web server was implemented for PDL1Binder, which is freely available at http://i.uestc.edu.cn/pdl1binder/cgi-bin/PDL1Binder.pl.

## Introduction

Blocking the immune checkpoint pathway is a highly promising therapeutic modality to fight cancer. Programmed cell death protein 1 (PD-1) is an immune checkpoint protein, which is mainly up-regulated on activated T cells, natural killer cells and B cells (Freeman et al., 2000). Programmed cell death ligand 1 (PD-L1) is a ligand for PD-1, which is highly expressed on many different malignancy cells and antigen-presenting cells (APCs) (Talantova et al., 2013). The interaction between PD-1 on T cells and PD-L1 on tumor cells leads to the inhibition of T-cell responses and loss of the cytotoxic T-cells’ functions and thereby mediates tumor cells to escape from the host immune surveillance. Blockade of this pathway can activate tumor-infiltrating T cells and restore their anti-tumor activity (Tumeh et al., 2014; Wolchok and Chan, 2014). Therefore, PD-1 and PD-L1 have become attractive therapeutic targets against cancer. Neoadjuvant anti-PD-1/PD-L1 therapy also achieved satisfactory clinical results in tumors (Li et al., 2021b).

Six PD-1/PD-L1 monoclonal antibody (mAb) blockers to date have been approved by FDA for cancer treatment (Postow et al., 2015; Robert et al., 2015; Bang et al., 2018; Tang et al., 2018). Moreover, most of PD-1/PD-L1 inhibitors in the clinical development are mAbs (Lin et al., 2020). Although mAbs targeting either PD-1 or PD-L1 have showed certain anti-tumor efficacy in cancer patients (Hamanishi et al., 2016), current mAb agents have many intrinsic limitations such as expensive production, still poor therapeutic responses (only approximately 20% of patients with a durable response) (Yang et al., 2016), and considerable individual differences as well as immunotherapy-induced improper immune-related responses (Fishman et al., 2019). Additionally, mAb therapeutics are accompanied by inevitable drawbacks including inferior organ or tumor penetration, poor oral bioavailability and immunogenicity. Compared to mAbs, peptides as drug candidates have several advantages, including higher tissue or tumor penetration, lower production costs and decreased immunogenicity. Peptides can also be subjected to chemical modifications to improve their pharmaceutical properties. However, PD-L1 binding peptides discovery through random peptide library screening is time consuming, expensive, and laborious.

In order to improve the efficiency of phage display selection, researchers have employed computational methods to aid analysis of results of random peptide library screening. For example, SAROTUP integrates a suite of tools which can be used to scan, report and exclude possible target-unrelated peptides from phage display biopanning results (He et al., 2019b). Sun et al. (2016) have proposed an epitope prediction method based on random peptide library screening. Machine learning methods have been used in mining and designing peptides of specific function (Tallorin et al., 2018; Ma et al., 2022). Obtaining therapeutic molecules is cheap and fast with the help of machine learning approaches (Liu et al., 2020; Laustsen et al., 2021). However, there are currently no bioinformatics tools to identify PD-L1 binding peptides.

Phage display permits high-throughput screening of peptide ligands with high affinity and specificity for almost any target of interest through several rounds of target-binding (selection) and amplification of phage display peptide libraries (Jaroszewicz et al., 2022; Ledsgaard et al., 2022). Moreover, phage display coupled with next-generation sequencing (NGPD) offers a more powerful tool to identify peptide ligands (Matochko and Derda, 2015; He et al., 2016, 2018a,2019c; Asar et al., 2020; Pleiko et al., 2021). Fewer biopanning rounds powered by deep sequencing can discover robust target-binding peptides that are not identified by Sanger sequencing (Juds et al., 2020). In addition, NGPD has been revealed very effective to suppress false-positive hits from amplification-induced bias (Matochko et al., 2014). Many researchers have employed traditional phage display technology to identify PD-L1 binding peptides (Li et al., 2018, 2021a; Liu et al., 2019; Tooyserkani et al., 2021), however, current existing PD-L1 binding peptides are not enough to implement a computational model for identifying PD-L1 binding peptides. NGPD can help to discover more novel PD-L1 binding peptide ligands. Illumina sequencing is a massively parallel sequencing technology and can produce large amounts of data (Quail et al., 2008). We screened the Ph.D.-12 phage display library against PD-L1, and here the selection output was investigated using Illumina sequencing.

In the present study, we aimed to develop a novel computational classifier for identifying peptides targeting PD-L1. We took advantage of NGPD to isolate PD-L1 binding peptides and used them to construct the predictive model *via* machine learning methods. First, we used PD-L1 as bait to screen the Ph.D.-12 phage display library. Second, the PD-L1 binding peptides isolated by phage display selection were paired with non-PD-L1 binding peptides. They were used to implement machine learning based models for predicting PD-L1 binding peptides. Third, we utilized two independent testing datasets to evaluate the generalization ability of the models. The PD-L1 binding peptides identified in this work hold high potential to be developed as anti-tumor therapeutics. The predictor for identifying peptides targeting PD-L1, called PDL1Binder, is valuable in accelerating PD-L1 binding peptides discovery and freely available at http://i.uestc.edu.cn/pdl1binder/cgi-bin/PDL1Binder.pl. Our study demonstrates that predictive models of PD-L1 binding can be learned from deep sequencing data and provides an efficient approach to discover PD-L1 binding peptides.

## Dataset and methods

### Phage display peptide library biopanning

We performed two rounds of phage display selection using recombinant human PD-L1 extracellular domain (ECD) protein (Cat# 10084-H08H, Sino Biological Inc., Beijing, China) as bait. The selection of Ph.D.-12 phage display library (New England Biolabs, Ipswitch, MA, United States) against PD-L1 was performed in six replicates. The control selections, i.e., Ph.D.-12 against Dynabeads (Cat# 10-103-D, Invitrogen) and Ph.D.-12 against unrelated anti-FLAG M2 monoclonal antibody (Cat# F3165, Sigma-Aldrich), were performed in triplicate.

### Round 1

In a microcentrifuge tube, 20 μL of Dynabeads were coated with a solution of PD-L1 (100 μL, 100 μg/mL) in PBS for overnight at 4 C. The solution was added to 900 μL of PBS and then transferred to a well in the KingFisher 96 deep-well plate (Cat# 95040450, Thermo Fisher Scientific). The Dynabeads with PD-L1 were rinsed 3 times with 0.1% Tween-20 in PBS, and then blocked with 2% (w/v) BSA in PBS for 1 h at room temperature, followed by an incubation with 3 × 10^11^ PFU Ph.D.-12 phage display library for 1.5 h at room temperature. The unbound phage was rinsed with 0.1% Tween-20 in PBS. Phage remained on the beads were eluted for 9 min at room temperature by adding 20 μL of HCl (pH 2). The elution buffer along with the beads were transferred into a 1.5 mL microcentrifuge tube and immediately neutralized with 10 μL of neutralization buffer (Phusion HF Buffer, NEB B0518S). The recovered phage was amplified in *E. coli* ER2738 (New England Biolabs, Ipswitch, MA, United States) for the second round of biopanning.

### Round 2

Six microcentrifuge tubes containing 20 μL of Dynabeads were coated with a solution of PD-L1 (100 μL, 100 μg/mL) in PBS for overnight at 4 C. An additional three microcentrifuge tubes containing 20 μL of Dynabeads were coated with a solution of Protein G (100 μg/mL) along with anti-FLAG M2 monoclonal antibody (150 μg/mL) in 100 μL PBS for overnight at 4 C. In parallel, three more microcentrifuge tubes containing 20 μL of Dynabeads were suspended in 100 μL PBS for overnight at 4 C. Solution from all 12 microcentrifuge tubes were then, respectively, added to 900 μL of PBS and transferred to 12 wells in a KingFisher 96 deep-well plate. The Dynabeads were rinsed with 0.1% Tween-20 in PBS, and then blocked with 2% (w/v) BSA in PBS for 1 h at room temperature, followed by incubation with 3 × 10^10^ PFU enriched Ph.D.-12 phage display library from Round 1 for 1.5 h at room temperature. The unbound phage was rinsed with 0.1% Tween-20 in PBS five times. Phage remained on the beads were resuspended in DNase free water and boiled at 90 C for 10 min. The single-stranded DNAs (ssDNAs) from discovered phage were extracted and subjected to polymerase chain reaction (PCR) amplification and Illumina sequencing, and those from Ph.D.-12 libraries before and after the first round of biopanning were also sequenced to serve as additional controls. The steps for Illumina sequencing of phage display libraries were described previously (He et al., 2018b). Briefly, PCR amplification was first performed to transform the ssDNA of the amplified phage into Illumina-compatible double-stranded DNA (dsDNA). The detailed PCR protocol for Illumina sequencing can be found in the Supplementary Information. After PCR amplification, the dsDNA PCR fragments corresponding to the expected size were confirmed and quantified using agarose gel electrophoresis. The PCR products from multiple experiments were then mixed together allowing 20 ng of each product in the mixture and purified by E-Gel (Thermo Fisher Scientific, Waltham, MA, United States). The purified dsDNAs were finally sequenced using the Illumina NextSeq paired-end 500/550 High Output Kit v2 (150 cycles).

### Deep-sequencing analysis

Raw FASTQ data were processed by using MatLab scripts described in a previous publication (He et al., 2018b) and filtered to find significantly enriched sequences using MatLab scripts previously reported on a computational server (Sugon I840-G20, Dawning Information Industry Co., LTD., Beijing, China). Sequences isolated from the PD-L1 screen that increased significantly in abundance against sequences isolated from the control selections were labeled PD-L1 binding peptides. Significance of the ratio was assessed using one-tailed, unequal variance Student *t*-test. Only sequences with ratio ≥ 2 and *p*-value ≤ 0.05 were considered as PD-L1 binding peptides. Deep sequencing the library before round 1 (R0), the output of two selection rounds (R1 and R2) and the control selection experiments identified 80 peptide sequences that exhibited high normalized abundance in R2 and low normalized abundance in R0, R1, and the control experiments R2-DB (Dynabeads), and R2-UF (unrelated anti-FLAG M2 monoclonal antibody).

### Database search for target-unrelated peptides

All sequences that were identified as potential PD-L1 binding peptides were searched against the BDB database^1^ (He et al., 2016) to check if they have been previously discovered in other phage display screens with distinct targets (MimoSearch^2^ and MimoScan^3^). Peptides that were identified by four or more entirely different targets were putative target-unrelated peptides (He et al., 2019a).

### Benchmark dataset for training

The positive dataset was composed of 80 PD-L1 binding peptides identified by NGPD. The remaining peptides were non-PD-L1 binding peptides, which consisted of the negative dataset. Redundant peptides were then removed by using CD-HIT (Li and Godzik, 2006; Fu et al., 2012), with a sequence identity threshold of 0.8 for the PD-L1 and non-PD-L1 binding peptides, respectively. We used this value based on the size of our dataset. More stringent criteria, such as 0.4 or 0.3, were not adopted because machine learning algorithms could not acquire abundant information to learn with a relatively small sample. After this analysis, no redundant peptides were found and excluded, and the positive training dataset consisted of 80 PD-L1 peptides. To balance the positive and negative training dataset, we randomly selected 800 peptides from the negative dataset and divided them into 10 sub-datasets. Each negative sub-dataset was paired with the positive training dataset. Finally, 10 pairs of sub-datasets were constructed and each pair was composed of 80 PD-L1 and 80 non-PD-L1 binding peptides (Table 1). The training dataset is provided in Trainingdataset.xslx in the Supplementary Material.

**TABLE 1:** Number of PD-L1 and non-PD-L1 binding peptides in each dataset.

Dataset	Number of PD-L1 binding peptides	Number of non-PD-L1 binding peptides
Training dataset	80	80/80/80/80/80/80/80/80/80/80
TestDataset_1	30	/
TestDataset_2	/	221405

*For the training dataset, each negative sub-dataset with 80 non-PD-L1 binding peptides was paired with the positive training dataset composed of 80 PD-L1 binding peptides.*

### Independent testing dataset construction

The literature data related to PD-L1 binding peptides were extracted from the PubMed database. A typical text mining query is given below: (anti-PD-L1 peptide) OR (PD-L1 binding peptide). The search returned 652 articles published before July 08, 2021. PD-L1 binding peptide sequences were then manually extracted from the above peer-reviewed papers. Modified peptides (peptides with non-natural amino acids) were first excluded since no modified peptides were in the training dataset. Inclusion criteria were as follows: (1) peptides containing only 20 natural amino acids and less than 50 residues were collected since peptides having more than 50 amino acids were considered as proteins; (2) peptides that have been experimentally verified to bind with PD-L1 *in vitro* or *in vivo* were collected. Finally, 34 experimentally validated PD-L1 binding peptides were obtained. After removing redundant peptides by using CD-HIT with a sequence identity cutoff of 0.8, 30 PD-L1 binding peptides were retained. Consequently, we constructed two independent testing datasets, i.e., TestDataset_1 (30 PD-L1 binding peptides) and TestDataset_2 [221405 non-PD-L1 binding peptides from the remaining negative dataset (not for training)] (Table 1). As the sources of the two datasets are different, they were tested separately. Datasets TestDataset_1 and TestDataset_2 are provided in Testingdataset.xlsx in the Supplementary Material.

### Sequence encoding and peptide descriptor analysis

Four peptide descriptors, including the amino acid composition (AAC), pseudo amino acid composition (PseAAC), dipeptide composition (DPC) and the composition of *k*-spaced amino acid group pairs (CKSAAGP), were used to encode each peptide in the training dataset. The calculation of the above descriptors were performed by the codes within the iLearnPlus (Chen et al., 2021). AAC and DPC are defined as follows:


(1) $$A\,A\,C\,\left(i\right)=\frac{x\,\left(i\right)}{\sum_{i=1}^{20}x\,\left(i\right)}$$

(2) $$D\,P\,C\,\left(j\right)=\frac{y\,\left(j\right)}{\sum_{j=1}^{400}y\,\left(j\right)}$$

where *AAC(i)* is the percent of the *ith* (*i* = 1, 2, …, 20) amino acid, and *x(i)* represents the number of the _*ith*_ amino acid in a peptide sequence. *DPC(j)* is the frequency of the *jth* (*j* = 1, 2, …, 400) dipeptide, and *y(j)* represents the number of the *jth* dipeptide in a peptide sequence.

The CKSAAGP descriptor is modified from the composition of *k*-spaced amino acid pairs (CKSAAP) in which the occurrences of the amino acid pairs that are separated by *k*-residues are calculated. For CKSAAGP, 20 amino acids are first divided into five groups according to their physicochemical properties: aromatic, aliphatic, negative-charged, positive-charged, and uncharged residues. The frequencies of the 25 amino acid pairs (5 × 5) separated by *k*-residues with group annotations were then calculated. For a peptide with *L* residues, if the *k*-spaced residue group pair *AE* appears *n* times, the frequency of the corresponding residue pair is *n*/[*L*−(*k* + 1)]. In this study, *k* = 0, 1 and 2 were jointly considered due to the peptide length of 12. Finally, the CKSAAGP descriptor with 25 × 3 = 75 dimensions was comprised of the frequencies of 0-spaced, 1-spaced, 2-spaced residue group pairs.

The sequence-order information would be completely ignored if AAC is used to encode a sequence. To compensate for AAC, PseAAC was proposed by introducing discrete factors for incorporating some sort of sequence-order or pattern information (Chou, 2001). The detailed calculation of PseAAC can be found at (Chou, 2009). In the formula for PseAAC, the weight factor ω and discrete counted-rank correlation factor λ are two key parameters. Considering the limited sequence length and to ensure the diversity of key components, we set λ = 4 and ω = 0.4 to generate PseAAC with 20 + λ dimensions.

### Feature selection

In this study, feature selection was implemented by using the iLearnPlus platform (Chen et al., 2021). Chi-square test (CHI2) (Forman, 2003), Information gain (IG) (Yu and Liu, 2003), F-score value (FScore) (Chen et al., 2018), Mutual information (MIC) (Peng et al., 2005), and Pearson’s correlation coefficient (Pearson) (Stigler, 1989) feature selection strategies were used to identify key features. The selected feature number was set to be 160 as each sub-dataset was comprised of 160 peptide sequences. The MinMax normalization approach was then utilized to scale the selected features to the unit range between 0 and 1. To select the optimal feature set, we further used various machine learning methods to construct models with each of the feature sets selected by CHI2, IG, FScore, MIC and Pearson feature selection approaches, respectively, *via* fivefold cross-validation. The feature set, which achieved the best classification performance, was utilized for further model construction.

### Machine learning algorithm selection

The optimal feature set obtained by feature selection was used to construct classifiers based on 12 state-of-the-art machine learning algorithms in the iLearnPlus-AutoML module *via* fivefold cross-validation (select the “Auto optimization” option to optimize parameters automatically), including Support vector machine (SVM) (Cortes and Vapnik, 1995), Random forest (RF) (Breiman, 2001), Decision tree (DecisionTree) (Breimann et al., 1984), K-nearest neighbors (KNN) (Altman, 1992), Logistic regression (LR) (Freedman, 2009), Gradient boosting decision tree (GBDT) (Friedman, 2001), Light gradient boosting machine (LightBGM) (Ke et al., 2017), Extreme gradient boosting (XGBoost) (Chen and Guestrin, 2016), Stochastic gradient descent (SGD) (Pedregosa et al., 2011), Naïve Bayes (NaïveBayes) (Rennie et al., 2003), Linear discriminant analysis (LDA) (McLachlan, 1992), and Quadratic discriminant analysis (QDA) (McLachlan, 1992).

### Performance evaluation

The fivefold cross-validation test was selected to evaluate the performance of the constructed classifiers. In the fivefold cross-validation test, the sequence dataset is randomly divided into five equally sized folds. Four folds of these folds are used to develop the machine learning model and optimize its parameters, and the remaining one fold is employed to assess the performance of the model. The process was repeated five times until each fold is used for testing once. In this study, eight commonly used metrics were utilized to quantify the model predictive performance, including sensitivity (Sn), specificity (Sp), Precision (Pr), F1 score (F1), accuracy (Acc), Matthews correlation coefficient (MCC), the area under the receiver operating characteristic (ROC) curve (AUROC) and the area under the precision-recall curve (AUPRC). The former six performance indicators are calculated by the following equations:


(3) $$S\,n=\frac{T\,P}{T\,P+F\,N}$$

(4) $$S\,p=\frac{T\,N}{T\,N+F\,P}$$

(5) $$P\,r=\frac{T\,P}{T\,P+F\,P}$$

(6) $$F\,1=\frac{2\times T\,P\times T\,P}{T\,P\times \left(T\,P+F\,N\right)+T\,P\times \left(T\,P+F\,P\right)}$$

(7) $$A\,c\,c=\frac{T\,P+T\,N}{T\,P+F\,P+T\,N+F\,N}$$

(8) $$M\,C\,C=\frac{T\,P\times T\,N-F\,P\times F\,N}{\sqrt{\left(T\,P+F\,P\right)\times \left(T\,P+F\,N\right)\times \left(T\,N+F\,P\right)\times \left(T\,N+F\,N\right)}}$$

where *TP*, *FP*, *TN*, *FN*, respectively, are the number of true positives, the number of the false positives, the number of true negatives, the number of the false negatives. We also computed the AUROC and AUPRC values for comparing the model performance.

### Final model construction and web service implementation

Ten submodels were constructed based on SVM by using the LIBSVM 3.25 package (Chang and Lin, 2011), which is available at http://www.csie.ntu.edu.tw/~cjlin/libsvm/. The radial basis function (RBF) was selected as the kernel function to develop SVM-based models. The kernel width factor *gamma* and the regularization factor *c* were automatically optimized by selecting the “Auto optimization” option *via* the grid search method in iLearnPlus (Chen et al., 2021). To reduce the generalization error of the prediction, we adopted the voting strategy to implement an ensemble predictor, called PDL1Binder. The ensemble model aggregates the predictive result of each submodel. In this study, we used the averaging voting technique, which takes an average of predictions from ten submodels and uses it to make the final prediction. Each peptide for prediction will be subjected to the prediction of ten submodels. Each submodel corresponds to a possibility value of the peptide being a PD-L1 binding peptide. The final probability value was computed by averaging the probability values of ten submodels. If the value is greater than or equal to the threshold of possibility value (0.5 by default), the peptide will be identified as a PD-L1 binding peptide.

For ease of use, the PDL1Binder classifier was further implemented into an online web service, which is freely available at http://i.uestc.edu.cn/pdl1binder/cgi-bin/PDL1Binder.pl. The web interface of PDL1Binder was developed by using Perl. The web service was tested in the Mozilla Firefox, Google Chrome, and Internet Explorer browsers.

## Results

**FIGURE 1:**
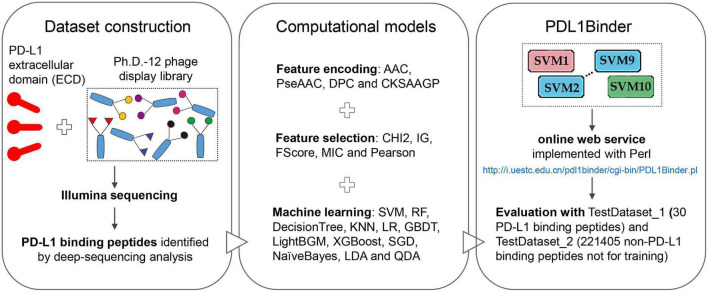
Overview of this study.

The workflow of this study is shown in Figure 1. We first isolated 80 PD-L1 binding peptides by using NGPD and utilized them as the benchmark dataset to develop computational models for identifying PD-L1 binding peptides. Four different peptide descriptors were employed to encode each peptide sequence. The optimal feature selection approach chosen from five feature selection strategies and 12 machine learning methods were combined to implement predictive models. Fivefold cross-validation results showed that the SVM-based model outperformed models developed with 11 other machine learning algorithms. Therefore, an ensemble SVM-based computational model, called PDL1Binder, was implemented. Moreover, two independent testing dataset: TestDataset_1 (30 PD-L1 binding peptides) and TestDataset_2 (221405 non-PD-L1 binding peptides not for training) were used to evaluate PDL1Binder.

### Selection and analysis of peptides that bind to programmed cell death ligand 1

We used the Ph.D.-12 phage display library to discover peptide ligands for PD-L1. Two rounds of phage display selection were performed using PD-L1 ECD as bait. In round 2, we also performed two control selections; in the first control, we panned the enriched Ph.D.-12 library from Round 1 against the Dynabeads (R2-DB) and in the second control, we panned the enriched Ph.D.-12 library from Round 1 against unrelated anti-FLAG M2 monoclonal antibody (R2-UF). Deep sequencing the library before round 1 (R0), the output of two selection rounds (R1 and R2) and the control selection experiments identified 80 peptide sequences that exhibited high normalized abundance in R2 and low normalized abundance in R0, R1, and the control experiments R2-DB, and R2-UF.

**FIGURE 2:**
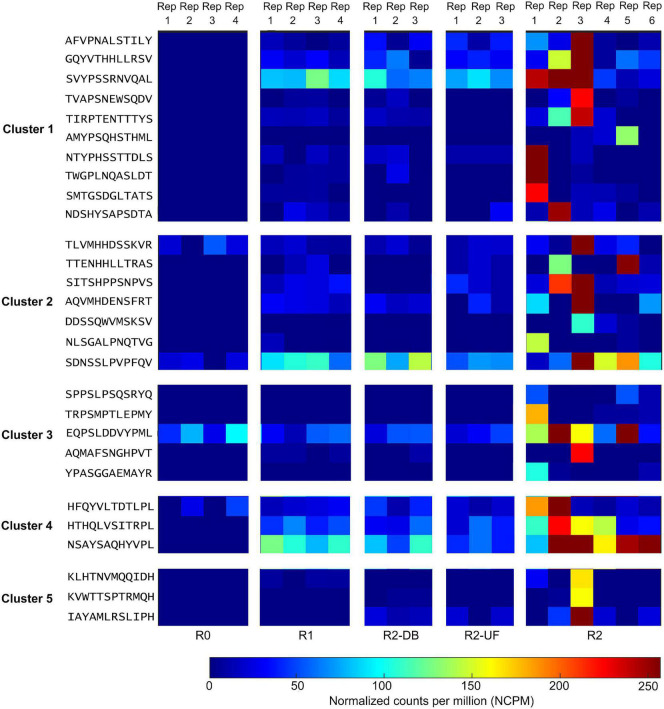
Deep sequencing the output of all selection rounds and the control experiments identified peptide sequences that exhibited high normalized abundance in R2 and low normalized abundance in R0, R1, and the control screens R2-DB, and R2-UF. Twenty-nine sequences from the deep sequencing results were clustered into five groups. Rep, replicate; R0, the library before round 1; R1, the first round of panning against PD-L1 ECD; R2-DB, panning the enriched Ph.D.-12 library from R1 against the Dynabeads; R2-UF, panning the enriched Ph.D.-12 library from R1 against unrelated anti-FLAG M2 monoclonal antibody (R2-UF); R2, panning the enriched Ph.D.-12 library from R1 against PD-L1 ECD.

All 80 potential peptide binders for PD-L1 were significantly enriched (*p* < 0.05, *R* ≥ 2) in the selection of the Ph.D.-12 phage display library on PD-L1 but not in any of the control screens (Supplementary Figure 1). We clustered the hit sequences based on their features described by the BLOSUM62 matrix and found that 29 peptides were clustered into five groups (Figure 2). The remaining un-clustered sequences were assigned to their nearest clusters (Supplementary Figure 1).

Finally, we investigated whether 80 PD-L1 binders could be target-unrelated peptides which are enriched for other reasons other than target specificity. MimoSearch and MimoScan confirmed that YPGSQSWMPSDF has been previously selected by IgE from patients, while the remaining 79 peptides have not been identified in other phage display biopanning datasets which are curated in BDB. As YPGSQSWMPSDF was only identified with two different targets so far, it was not considered as a target-unrelated peptide and remained for further analysis.

### Performance analysis of models trained with diverse machine learning and feature selection methods

The AAC, DPC, CKSAAGP, and PseAAC descriptors were used to encode each peptide in the training dataset. We directly concatenated four types of peptide descriptors. As a result, the dimension of the feature vector of each peptide is 519 (Table 2). For each of the ten sub-datasets, feature selection was performed by using five popular feature selection approaches, respectively. The number of selected features was determined to be 160 to keep the same as the number of peptides in the training dataset.

**TABLE 2:** List of 519 features.

Peptide descriptor	Feature dimension
Amino acid composition (AAC)	20
Dipeptide composition (DPC)	400
Pseudo amino acid composition (PseAAC)	24
Composition of *k*-spaced amino acid group pairs (CKSAAGP)	75
(AAC, DPC, PseAAC, CKSAAGP)	519

*Each peptide was represented by four types of peptide descriptors, which were conflated into a feature vector with 519 dimensions.*

**FIGURE 3:**
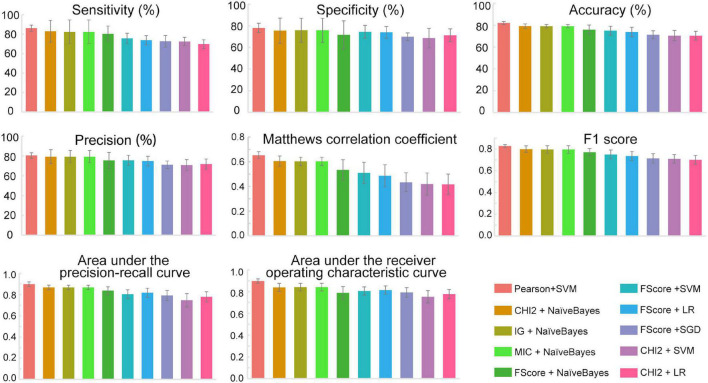
The performance metrics of each submodel. All data were expressed as mean ± standard deviation. SVM, Support vector machine; LR, Logistic regression; SGD, Stochastic gradient descent; NaïveBayes, Naïve Bayes; Pearson, Pearson’s correlation coefficient; CHI2, Chi-square test; IG, Information gain; FScore, F-score value; MIC, Mutual information.

The feature subsets obtained through various feature selection methods were then used to develop predictors with 12 different traditional machine learning methods implemented in iLearnPlus (Chen et al., 2021). As shown in Figure 3, the results of fivefold cross-validation showed that the SVM-based classifier trained with the feature set selected by Pearson’s correlation coefficient achieved an average accuracy of 82.13% with an average of 86.13% sensitivity and 78.13% specificity. For all ten submodels, the AUROC and AUPRC values of the SVM-based model are the highest. The model under this combination outperformed other models developed with different feature subsets and machine learning algorithms (see Machinelearningresult.xlsx in Supplementary Material). Therefore, feature subsets selected by Pearson’s correlation coefficient and SVM were utilized for further model construction. Performance metrics of each submodel under each combination were provided in Machinelearningresult.xlsx in Supplementary Material.

### Ensemble predictor for identifying programmed cell death ligand 1 binding peptides

**FIGURE 4:**
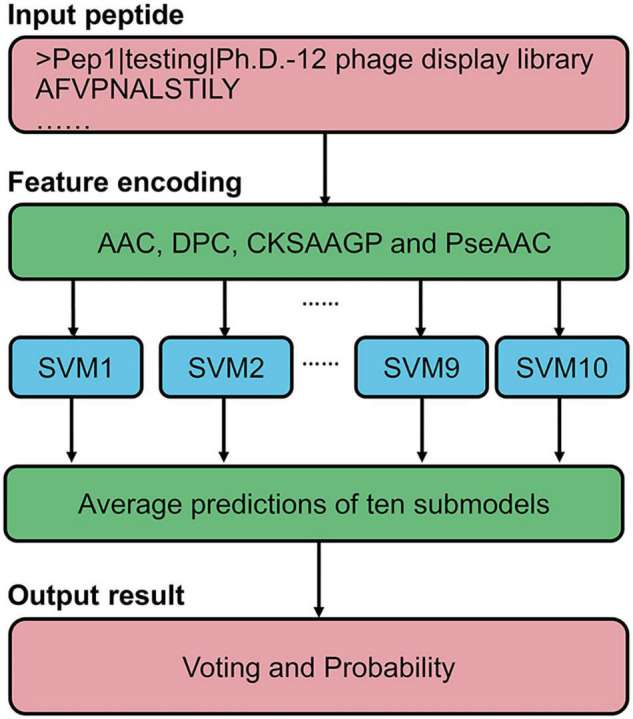
Framework of the proposed scheme for PD-L1 binding peptide prediction.

Based on the above results, we proposed an ensemble SVM-based predictor for identifying PD-L1 binding peptides, called PDL1Binder, the framework of which is illustrated in Figure 4. For a given peptide, it will be predicted by ten submodels separately. PDL1Binder then uses the averaging voting method and makes final predictions based on the average probability value. The fivefold cross-validation results in Figure 3 showed that PDL1Binder achieved an average accuracy of 82.13% with an average of 86.13% sensitivity, 80.42% precision, and 78.13% specificity, and an average of 0.6528 MCC, 0.8271 F1, 0.8978 AUROC, and 0.8989 AUPRC.

**FIGURE 5:**
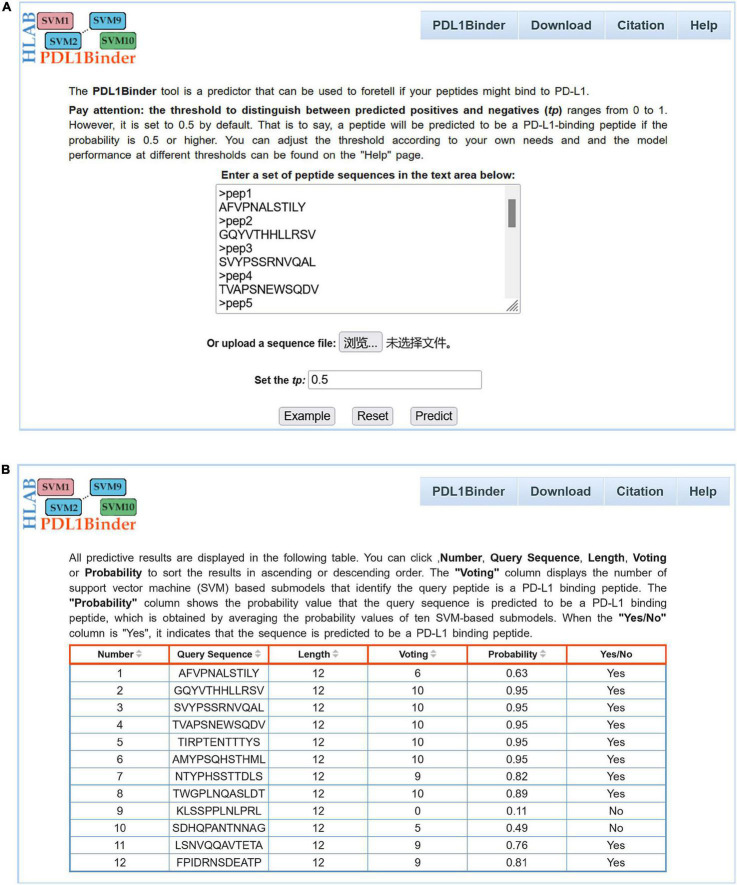
Webpage of PDL1Binder. **(A)** Input interface of PDL1Binder. Users can submit query sequences in FASTA or plain text format. The *tp* can be set by users, ranging from 0 to 1. **(B)** Output interface of PDL1Binder. PDL1Binder outputs the number of SVM-based submodels that identify the query peptide is a PD-L1 binding peptide and the probability value that the query sequence is predicted to be a PD-L1 binding peptide. The output likelihood value is obtained by averaging the probability values of 10 SVM-based submodels.

For the convenience of users in using PDL1Binder, an online web service has been developed, which is freely available at http://i.uestc.edu.cn/pdl1binder/cgi-bin/PDL1Binder.pl. As shown in Figure 5, a professional and user-friendly web architecture for PDL1Binder was implemented. PDL1Binder allows users to submit peptide sequences in fasta or plain text format and set the threshold of probability value to differentiate between predicted positives and negatives (*tp*) (Figure 5A), which makes it more convenient and flexible for future users. To simplify the representation of PDL1Binder prediction, predictive results are displayed in a table (Figure 5B). Users can sort the results in ascending or descending order by a specific column.

### Evaluation of PDL1Binder with independent testing datasets

Two independent testing datasets, one with 30 non-redundant PD-L1 binding peptides and the other one with 221405 non-redundant non-PD-L1 binding peptides, were employed to evaluate the generalization ability of PDL1Binder under different *tp*-values. As shown in Table 3, with the increase of the *tp*-value, the value of sensitivity decreases, while the specificity value increases. When the *tp*-value was set to 0.55 within PDL1Binder, 83.33% PD-L1 binding peptides in the TestDataset_1 were correctly identified as PD-L1 binding peptides, while 53.29% non-PD-L1 binding peptides were precisely predicted as non-PD-L1 binding peptides in the TestDataset_2 (Table 3).

**TABLE 3:** Performance of PDL1Binder in two independent testing datasets under different *tp*-values.

*tp*	0.1	0.15	0.20	0.25	0.30	0.35
TestDataset_1	100.00%	100.00%	100.00%	96.67%	93.33%	93.33%
TestDataset_2	0.80%	2.79%	5.03%	8.92%	15.18%	20.29%
*tp*	0.40	0.45	0.50	**0.55**	0.60	0.65
TestDataset_1	93.33%	86.67%	83.33%	**83.33%**	63.33%	53.33%
TestDataset_2	27.52%	37.39%	44.31%	**53.29%**	62.34%	71.17%
*tp*	0.70	0.75	0.80	0.85	0.90	0.95
TestDataset_1	43.33%	43.33%	30.00%	10.00%	3.33%	0.00%
TestDataset_2	80.84%	86.43%	92.26%	96.52%	99.09%	100.00%

*tp, threshold of probability value to differentiate between predicted positives and negatives. Performance metric is the predictive accuracy of PD-L1 binding for TestDataset_1 and that of non-PD-L1 binding for TestDataset_2. Bold: The predictive accuracy of PD-L1 binding and that of non-PD-L1 binding have reached their maximum under tp = 0.55.*

## Discussion

Many studies have demonstrated that PD-L1 binding peptides are promising for the treatment of cancers (Pan et al., 2021). PD-L1 binding peptides screened by phage display selection in this study could serve as peptide drug candidates for blocking the PD-1/PD-L1 interaction. However, only a few tens of PD-L1 binding peptides have been experimentally identified. In fact, many candidate molecules are needed to develop a peptide drug for cancer immunotherapy. Therefore, it is urgently needed to employ computational methods to rapidly identify more novel PD-L1 binding peptides.

At present, no computational models have been proposed for efficiently discovering PD-L1 binding peptides. To pursue identifying PD-L1 binding peptides from pools of peptides with unknown functions, we designed a SVM-based classifier based on sequence information, called PDL1Binder, which could help to eliminate false positive peptides and improve the efficiency of obtaining PD-L1 binding peptides. The classifier integrates 10 SVM submodels. Two independent testing datasets were constructed to test the performance of PDL1Binder. Here, 83.33% of PD-L1 binding peptides in the TestDataset_1 were correctly identified as PD-L1 binding peptides, while 53.29% of non-PD-L1 binding peptides were precisely predicted as non-PD-L1 binding peptides in the TestDataset_2 when the *tp*-value was set to 0.55 within PDL1Binder. The proposed approach is considered as an applicable scheme for assisting the development of novel PD-L1 binding peptides.

PDL1Binder could be beneficial for both panning experiments and subsequent affinity determination experiments. The model can help researchers remove as many non-PD-L1 binding peptides as possible, thereby reducing both time and costs involved in getting PD-L1 binding peptide candidates. After testing on two independent test sets, we found that PDL1Binder was able to help remove around 5% of non-PD-L1 binding peptides (TestDataset_2) while retaining almost all PD-L1 binding peptides (TestDataset_1) (*tp* = 0.2). When the tp-value was set to 0.5, PDL1Binder correctly predicted 83.33% of PD-L1 binding peptides (TestDataset_1) while clearing away 44.31% of non-PD-L1 binding peptides (TestDataset_2). Additionally, our tool could successfully eliminate more than half of non-PD-L1 binding peptides (53.29%, TestDataset_2) while reserving 83.33% of PD-L1 binding peptides (TestDataset_1) (*tp* = 0.55). In the actual situation of random peptide library screening experiment, PD-L1 binding peptides are fewer and more precious, so researchers wish to keep as many putative PD-L1 binding peptides as possible in an experiment, while the proportion of non-PD-L1 binding peptides is much larger than that of PD-L1 binding peptides, thereby they wish to remove as many non-PD-L1 binding peptides as possible. We recommend users to set *tp* at 0.55 when using PDL1Binder since both the predictive accuracy of PD-L1 binding and that of non-PD-L1 binding have reached their maximum under this threshold. The above results indicate that PDL1Binder might save a significant amount of time and cost, greatly improving the efficiency of discovering PD-L1 binding peptides.

In the process of removing redundant peptides from 80 PD-L1 binding peptides identified by phage display screen, no redundant peptides were found and excluded. This suggests that these PD-L1 binding peptides seem to have a low sequence identity (below 0.8), which indicates that there are fewer features that are consistent within the PD-L1 binding sequences in the low-dimensional space. The SVM algorithm first projects the features in a low-dimensional space to those in a high-dimensional feature space through the RBF kernel function, and more consistent features are found in the high-dimensional feature space. We speculate that this might be a reason why SVM is superior to other machine learning algorithms. Another possible reason might be that LIBSVM utilizes L1 regularization (Chang and Lin, 2011), which could effectively avoid overfitting on a small training dataset. SVM with RBF kernel (RBFSVM) can handle the overfitting problem through selecting appropriate kernel width factor *gamma* and regularization factor *c*.

Our dataset for training is relatively small. More experimentally validated PD-L1 binding peptides will be needed to improve the performance of the computational model for identifying PD-L1 binding peptides. In the future, we will continue to improve the model and synthesize potential PD-L1 binding peptides predicted by the model to experimentally show if they can bind with PD-L1.

## Conclusion

PD-L1 binding peptides are potential therapeutic agents for treating cancers. The PD-L1 binding peptides identified by phage display screen in this study are promising to become peptide drug candidates for blocking the PD-1/PD-L1 interaction to combat cancer. Computational models for identifying PD-L1 binding peptides can accelerate the discovery of these novel drug candidates. This study proposes the first SVM-based computational model, PDL1Binder, for effectively predicting peptides targeting PD-L1. We implemented PDL1Binder into an online web-server, which is freely accessible at http://i.uestc.edu.cn/pdl1binder/cgi-bin/PDL1Binder.pl. Our study showcases the potential of machine learning approaches for mining PD-L1 binding peptides from peptide pools of unknown bioactivities and provides promising PD-L1 binding peptide candidates for in-depth investigations.

## Data Availability Statement

The original contributions presented in this study are included in the article/Supplementary Material, further inquiries can be directed to the corresponding author/s.

## Author contributions

JH carried out the concept and design of this study. BH was responsible for data acquisition, constructed models, and drafted the manuscript. BL constructed models and prepared the manuscript for submission. QZ, BL, and CL repeated the model construction. SY and JL prepared the figures and tables. LN and HC guided modeling. All authors contributed to manuscript revision.

## Conflict of Interest

The authors declare that the research was conducted in the absence of any commercial or financial relationships that could be construed as a potential conflict of interest.

## Publisher’s Note

All claims expressed in this article are solely those of the authors and do not necessarily represent those of their affiliated organizations, or those of the publisher, the editors and the reviewers. Any product that may be evaluated in this article, or claim that may be made by its manufacturer, is not guaranteed or endorsed by the publisher.

## Footnotes

1
http://i.uestc.edu.cn/bdb/

2
http://i.uestc.edu.cn/bdb/index.php/site/tools?type=MimoSearch

3
http://i.uestc.edu.cn/bdb/index.php/site/tools?type=MimoScan

## References

[B1] Altman N. S. (1992). An introduction to kernel and nearest-neighbor nonparametric regression. *Am. Stat.* 46 175–185. 10.2307/2685209

[B2] Asar M. C. Franco A. Soendergaard M. (2020). Phage display selection, identification, and characterization of novel pancreatic cancer targeting peptides. *Biomolecules* 10:714. 10.3390/biom10050714 32380649 PMC7277971

[B3] Bang Y. J. Ruiz E. Y. Van Cutsem E. Lee K. W. Wyrwicz L. Schenker M. (2018). Phase III, randomised trial of avelumab versus physician’s choice of chemotherapy as third-line treatment of patients with advanced gastric or gastro-oesophageal junction cancer: primary analysis of JAVELIN Gastric 300. *Ann. Oncol.* 29 2052–2060. 10.1093/annonc/mdy264 30052729 PMC6225815

[B4] Breiman L. (2001). Random forests. *Mach. Learn.* 45 5–32. 10.1023/A:1010933404324

[B5] Breimann L. Friedman J. H. Olshen R. A. Stone C. J. (1984). *Classification and Regression Trees.* New York, NY: Taylor & Francis.

[B6] Chang C.-C. Lin C.-J. (2011). LIBSVM: a library for support vector machines. *ACM Transac. Intel. Syst. Technol.* 2 1–27. 10.1145/1961189.1961199

[B7] Chen J. Guo M. Wang X. Liu B. (2018). A comprehensive review and comparison of different computational methods for protein remote homology detection. *Brief Bioinform.* 19 231–244. 10.1093/bib/bbw108 27881430

[B8] Chen T. Guestrin C. (2016). “Xgboost: A scalable tree boosting system,” in *Proceedings of the 22nd ACM Sigkdd International Conference on Knowledge Discovery and Data Mining*, New York, NY.

[B9] Chen Z. Zhao P. Li C. Li F. Xiang D. Chen Y. Z. (2021). iLearnPlus: a comprehensive and automated machine-learning platform for nucleic acid and protein sequence analysis, prediction and visualization. *Nucleic Acids Res.* 49 e60. 10.1093/nar/gkab122 33660783 PMC8191785

[B10] Chou K.-C. (2009). Pseudo Amino Acid Composition and its Applications in Bioinformatics, Proteomics and System Biology. *Curr. Prot.* 6 262–274. 10.2174/157016409789973707

[B11] Chou K. C. (2001). Prediction of protein cellular attributes using pseudo-amino acid composition. *Proteins* 43 246–255. 10.1002/prot.1035 11288174

[B12] Cortes C. Vapnik V. (1995). Support-vector networks. *Mach. Learn.* 20 273–297. 10.1007/BF00994018

[B13] Fishman J. A. Hogan J. I. Maus M. V. (2019). Inflammatory and infectious syndromes associated with cancer immunotherapies. *Clin. Infect. Dis.* 69 909–920. 10.1093/cid/ciy1025 30520987

[B14] Forman G. (2003). An extensive empirical study of feature selection metrics for text classification. *J. Mach. Learn. Res.* 3 1289–1305. 10.1162/153244303322753670

[B15] Freedman D. A. (2009). *Statistical Models: Theory and Practice.* Cambridge, MA: Cambridge University Press.

[B16] Freeman G. J. Long A. J. Iwai Y. Bourque K. Chernova T. Nishimura H. (2000). Engagement of the PD-1 immunoinhibitory receptor by a novel B7 family member leads to negative regulation of lymphocyte activation. *J. Exp. Med.* 192 1027–1034. 10.1084/jem.192.7.1027 11015443 PMC2193311

[B17] Friedman J. H. (2001). Greedy function approximation: a gradient boosting machine. *Ann. Stat.* 29 1189–1232. 10.2307/2699986

[B18] Fu L. Niu B. Zhu Z. Wu S. Li W. (2012). CD-HIT: accelerated for clustering the next-generation sequencing data. *Bioinformatics* 28, 3150–3152. 10.1093/bioinformatics/bts565 23060610 PMC3516142

[B19] Hamanishi J. Mandai M. Matsumura N. Abiko K. Baba T. Konishi I. (2016). PD-1/PD-L1 blockade in cancer treatment: perspectives and issues. *Int. J. Clin. Oncol.* 21 462–473. 10.1007/s10147-016-0959-z 26899259 PMC4901122

[B20] He B. Chai G. Duan Y. Yan Z. Qiu L. Zhang H. (2016). BDB: biopanning data bank. *Nucleic Acids Res.* 44 D1127–D1132. 10.1093/nar/gkv1100 26503249 PMC4702802

[B21] He B. Chen H. Huang J. (2019a). PhD7Faster 2.0: predicting clones propagating faster from the Ph.D.-7 phage display library by coupling PseAAC and tripeptide composition. *PeerJ* 7:e7131. 10.7717/peerj.7131 31245183 PMC6585900

[B22] He B. Chen H. Li N. Huang J. (2019b). SAROTUP: a suite of tools for finding potential target-unrelated peptides from phage display data. *Int. J. Biol. Sci.* 15 1452–1459. 10.7150/ijbs.31957 31337975 PMC6643146

[B23] He B. Dzisoo A. M. Derda R. Huang J. (2019c). Development and application of computational methods in phage display technology. *Curr. Med. Chem.* 26 7672–7693. 10.2174/0929867325666180629123117 29956612

[B24] He B. Jiang L. Duan Y. Chai G. Fang Y. Kang J. (2018a). Biopanning data bank 2018: hugging next generation phage display. *Database* 2018:bay032. 10.1093/database/bay032 29688378 PMC7206649

[B25] He B. Tjhung K. F. Bennett N. J. Chou Y. Rau A. Huang J. (2018b). Compositional bias in naive and chemically-modified phage-displayed libraries uncovered by paired-end deep sequencing. *Sci. Rep.* 8:1214. 10.1038/s41598-018-19439-2 29352178 PMC5775325

[B26] Jaroszewicz W. Morcinek-Orlowska J. Pierzynowska K. Gaffke L. Wegrzyn G. (2022). Phage display and other peptide display technologies. *FEMS Microbiol. Rev.* 46:fuab052. 10.1093/femsre/fuab052 34673942

[B27] Juds C. Schmidt J. Weller M. G. Lange T. Beck U. Conrad T. (2020). Combining Phage Display and Next-Generation Sequencing for Materials Sciences: a Case Study on Probing Polypropylene Surfaces. *J. Am. Chem. Soc.* 142 10624–10628. 10.1021/jacs.0c03482 32460497

[B28] Ke G. Meng Q. Finley T. Wang T. Chen W. Ma W. (2017). “Lightgbm: A highly efficient gradient boosting decision tree,” in *Proceedings of the 31st International Conference on Neural Information Processing Systems*, Red Hook, NY. 10.5555/3294996.3295074

[B29] Laustsen A. H. Greiff V. Karatt-Vellatt A. Muyldermans S. Jenkins T. P. (2021). Animal immunization, *in vitro* display technologies, and machine learning for antibody discovery. *Trends Biotechnol.* 39 1263–1273. 10.1016/j.tibtech.2021.03.003 33775449

[B30] Ledsgaard L. Ljungars A. Rimbault C. Sorensen C. V. Tulika T. Wade J. (2022). Advances in antibody phage display technology. *Drug Discov. Today* 27 2151–2169. 10.1016/j.drudis.2022.05.002 35550436

[B31] Li W. Godzik A. (2006). Cd-hit: a fast program for clustering and comparing large sets of protein or nucleotide sequences. *Bioinformatics* 22, 1658–1659. 10.1093/bioinformatics/btl158 16731699

[B32] Li C. Zhang N. Zhou J. Ding C. Jin Y. Cui X. (2018). Peptide Blocking of PD-1/PD-L1 Interaction for Cancer Immunotherapy. *Cancer Immunol. Res.* 6 178–188. 10.1158/2326-6066.CIR-17-0035 29217732

[B33] Li Z. Wu X. Zhao Y. Xiao Y. Zhao Y. Zhang T. (2021b). Clinical benefit of neoadjuvant anti-PD-1/PD-L1 utilization among different tumors. *MedComm* 2 60–68. 10.1002/mco2.61 34766136 PMC8491227

[B34] Li W. Zhu X. Zhou X. Wang X. Zhai W. Li B. (2021a). An orally available PD-1/PD-L1 blocking peptide OPBP-1-loaded trimethyl chitosan hydrogel for cancer immunotherapy. *J. Control Release* 334 376–388. 10.1016/j.jconrel.2021.04.036 33940058

[B35] Lin X. Lu X. Luo G. Xiang H. (2020). Progress in PD-1/PD-L1 pathway inhibitors: from biomacromolecules to small molecules. *Eur. J. Med. Chem.* 186 111876. 10.1016/j.ejmech.2019.111876 31761384

[B36] Liu G. Zeng H. Mueller J. Carter B. Wang Z. Schilz J. (2020). Antibody complementarity determining region design using high-capacity machine learning. *Bioinformatics* 36 2126–2133. 10.1093/bioinformatics/btz895 31778140 PMC7141872

[B37] Liu H. Zhao Z. Zhang L. Li Y. Jain A. Barve A. (2019). Discovery of low-molecular weight anti-PD-L1 peptides for cancer immunotherapy. *J. Immunother. Cancer* 7:270. 10.1186/s40425-019-0705-y 31640814 PMC6805442

[B38] Ma Y. Guo Z. Xia B. Zhang Y. Liu X. Yu Y. (2022). Identification of antimicrobial peptides from the human gut microbiome using deep learning. *Nat. Biotechnol.* 40 921–931. 10.1038/s41587-022-01226-0 35241840

[B39] Matochko W. L. Cory Li S. Tang S. K. Derda R. (2014). Prospective identification of parasitic sequences in phage display screens. *Nucleic Acids Res.* 42 1784–1798. 10.1093/nar/gkt1104 24217917 PMC3919620

[B40] Matochko W. L. Derda R. (2015). Next-generation sequencing of phage-displayed peptide libraries. *Methods Mol. Biol.* 1248 249–266. 10.1007/978-1-4939-2020-4_17 25616338

[B41] McLachlan G. (1992). *Discriminant Analysis and Statistical Pattern Recognition.* New York, NY: John Wiley & Sons.

[B42] Pan C. Yang H. Lu Y. Hu S. Wu Y. He Q. (2021). Recent advance of peptide-based molecules and nonpeptidic small-molecules modulating PD-1/PD-L1 protein-protein interaction or targeting PD-L1 protein degradation. *Eur. J. Med. Chem.* 213:113170. 10.1016/j.ejmech.2021.113170 33454550

[B43] Pedregosa F. Varoquaux G. Gramfort A. Michel V. Thirion B. Grisel O. (2011). Scikit-learn: machine learning in Python. *J. Mach. Learn. Res.* 12 2825–2830. 10.48550/arXiv.1201.0490

[B44] Peng H. Long F. Ding C. (2005). Feature selection based on mutual information: criteria of max-dependency, max-relevance, and min-redundancy. *IEEE Trans. Pattern Anal. Mach. Intell.* 27 1226–1238. 10.1109/TPAMI.2005.159 16119262

[B45] Pleiko K. Posnograjeva K. Haugas M. Paiste P. Tobi A. Kurm K. (2021). *In vivo* phage display: identification of organ-specific peptides using deep sequencing and differential profiling across tissues. *Nucleic Acids Res.* 49:e38. 10.1093/nar/gkaa1279 33444445 PMC8053097

[B46] Postow M. A. Chesney J. Pavlick A. C. Robert C. Grossmann K. McDermott D. (2015). Nivolumab and ipilimumab versus ipilimumab in untreated melanoma. *N. Engl. J. Med.* 372 2006–2017. 10.1056/NEJMoa1414428 25891304 PMC5744258

[B47] Quail M. A. Kozarewa I. Smith F. Scally A. Stephens P. J. Durbin R. (2008). A large genome center’s improvements to the Illumina sequencing system. *Nat. Methods* 5 1005–1010. 10.1038/nmeth.1270 19034268 PMC2610436

[B48] Rennie J. D. Shih L. Teevan J. Karger D. R. (2003). “Tackling the poor assumptions of naive bayes text classifiers,” in *Proceedings of the 20th international conference on machine learning (ICML-03)*, Washington, DC. 10.5555/3041838.3041916

[B49] Robert C. Schachter J. Long G. V. Arance A. Grob J. J. Mortier L. (2015). Pembrolizumab versus Ipilimumab in Advanced Melanoma. *N. Engl. J. Med.* 372 2521–2532. 10.1056/NEJMoa1503093 25891173

[B50] Stigler S. M. (1989). Francis Galton’s account of the invention of correlation. *Stat. Sci.* 4 73–79. 10.1214/ss/1177012580

[B51] Sun P. Qi J. Zhao Y. Huang Y. Yang G. Ma Z. (2016). A novel conformational B-cell epitope prediction method based on mimotope and patch analysis. *J. Theor. Biol.* 394 102–108. 10.1016/j.jtbi.2016.01.021 26804644

[B52] Talantova M. Sanz-Blasco S. Zhang X. Xia P. Akhtar M. W. Okamoto S. (2013). Aβ induces astrocytic glutamate release, extrasynaptic NMDA receptor activation, and synaptic loss. *Proc. Natl. Acad. Sci. U.S.A.* 110 E2518–E2527. 10.1073/pnas.1306832110 23776240 PMC3704025

[B53] Tallorin L. Wang J. Kim W. E. Sahu S. Kosa N. M. Yang P. (2018). Discovering de novo peptide substrates for enzymes using machine learning. *Nat. Commun.* 9:5253. 10.1038/s41467-018-07717-6 30531862 PMC6286390

[B54] Tang J. Yu J. X. Hubbard-Lucey V. M. Neftelinov S. T. Hodge J. P. Lin Y. (2018). Trial watch: the clinical trial landscape for PD1/PDL1 immune checkpoint inhibitors. *Nat. Rev. Drug Discov.* 17 854–855. 10.1038/nrd.2018.210 30482962

[B55] Tooyserkani R. Rasaee M. J. Bandehpour M. W P M Löwik D. (2021). Novel anti-PD-L1 peptide selected from combinatorial phage library inhibits tumor cell growth and restores T-cell activity. *J. Drug Target* 29 771–782. 10.1080/1061186X.2021.1879087 33478285

[B56] Tumeh P. C. Harview C. L. Yearley J. H. Shintaku I. P. Taylor E. J. Robert L. (2014). PD-1 blockade induces responses by inhibiting adaptive immune resistance. *Nature* 515 568–571. 10.1038/nature13954 25428505 PMC4246418

[B57] Wolchok J. D. Chan T. A. (2014). Cancer: antitumour immunity gets a boost. *Nature* 515 496–498. 10.1038/515496a 25428495 PMC6592276

[B58] Yang C. Y. Lin M. W. Chang Y. L. Wu C. T. Yang P. C. (2016). Programmed cell death-ligand 1 expression is associated with a favourable immune microenvironment and better overall survival in stage I pulmonary squamous cell carcinoma. *Eur. J. Cancer* 57 91–103. 10.1016/j.ejca.2015.12.033 26901614

[B59] Yu L. Liu H. (2003). “Feature selection for high-dimensional data: A fast correlation-based filter solution,” in *Proceedings of the 20th international conference on machine learning (ICML-03)*, Washington, DC. 10.5555/3041838.3041946

