# Supplementary material for: PDL1Binder: Identifying programmed cell death ligand 1 binding peptides by incorporating next-generation phage display data and different peptide descriptors
Source: Front Microbiol. 2022 Jul 15;13:928774. doi: 10.3389/fmicb.2022.928774 (PMC9335124; doi:10.3389/fmicb.2022.928774)
Supplement: Supplementary file 4 [file Data_Sheet_4.pdf]

Supplementary Information for:

PDL1Binder: identifying PD-L1 binding peptides by incorporating next-generation phage display data and different peptide descriptors

Bifang He[1], Bowen Li[1], Xue Chen[1], Qianyue Zhang[1], Chunying Lu[1], Shanshan Yang[1], Jinjin Long[1], Lin Ning[2], Heng Chen[1,*], Jian Huang[3,*]

[1] Medical College, Guizhou University, Guiyang 550025, Guizhou Province, China

[2] School of Healthcare Technology, Chengdu Neusoft University, Chengdu, Sichuan Province, China

[3] School of Life Science and Technology, University of Electronic Science and Technology of China, Chengdu 611731, Sichuan Province, China

[*] Correspondence should be addressed to Jian Huang (email: hj@uestc.edu.cn) or Heng Chen (email: hchen13@gzu.edu.cn).

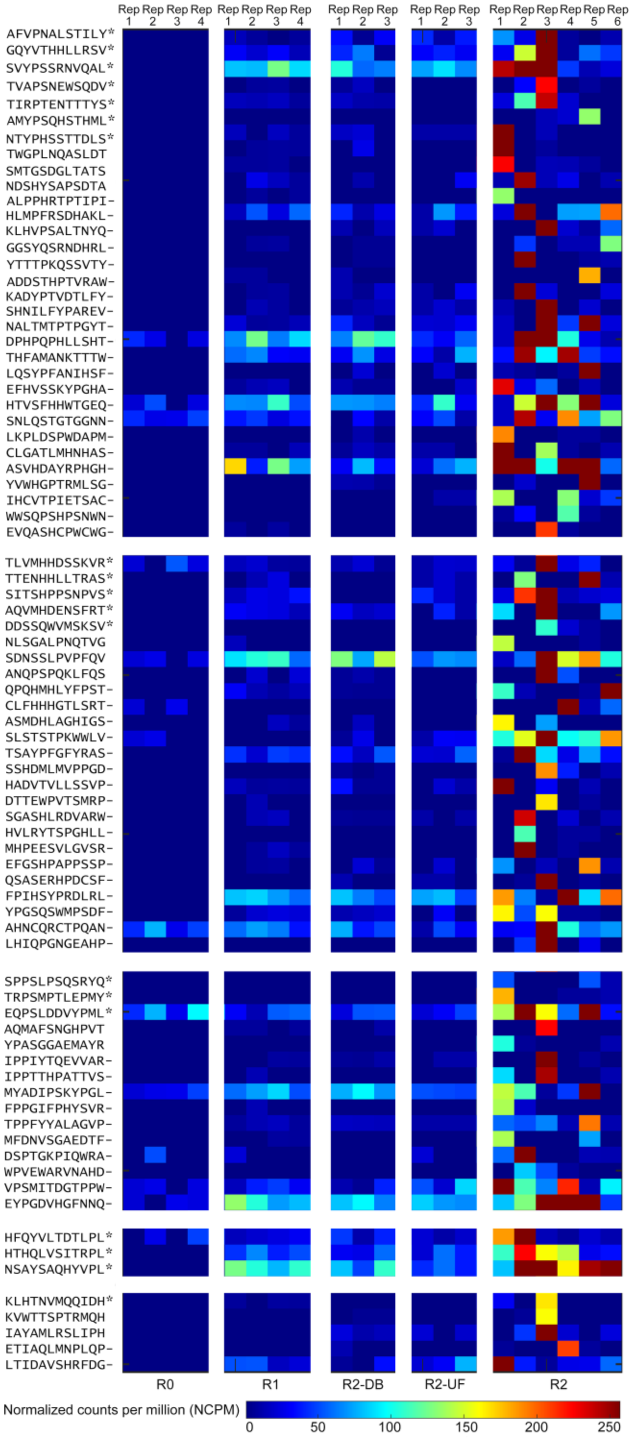

Figure S1 Deep sequencing the output of all selection rounds and the control experiments identified 80 peptide sequences that exhibited high normalized abundance in R2 and low normalized abundance in R0, R1, and the control screens R2-DB, and R2-UF. Twenty-nine sequences from the deep sequencing results were clustered into five groups. * core sequence in the cluster, - unclustered sequence, but close to the cluster than any other clusters, Rep: replicate, R0: the library before round 1, R1: the first round of panning against PD-L1 ECD, R2-DB: panning the enriched Ph.D.-12 library from R1 against the Dynabeads, R2-UF: panning the enriched Ph.D.-12 library from R1 against unrelated anti-FLAG M2 monoclonal antibody (R2-UF), R2: panning the enriched Ph.D.-12 library from R1 against PD-L1 ECD.

1 PCR protocol for Illumina sequencing

Briefly, single-stranded DNA (ssDNA) of the amplified phage was extracted using QIAprep spin M13 kit (Qiagen, #27704) according to manufacturer's instructions. The ssDNA was then transformed into Illumina-compatible double-stranded DNA (dsDNA) by PCR amplification using forward and reverse primers which were provided as follows: forward primer: 5'-CAAGCAGAAGACGGCATACGAGATCGGTCTCGGCATTCCTGCTGAACCGCTCTTCCGATCTXXXXCCTTTCTATTCTCACTCT-3' and reverse primer: 5'-AATGATACGGCGACCACCGAGATCTACACTCTTTCCCTACACGACGCTCTTCCGATCTXXXXACAGTTTCGGCCGA-3'. The XXXX in the primer sequence represents different indexing barcodes which were used to track distinct samples in an Illumina sequencing experiment. The extracted ssDNA was amplified in a 50 μL PCR reaction mixture, containing 1× Phusion® buffer (NEB), 1 mM each dNTPs, 0.5 μM forward barcoded primer, 0.5 μM reverse barcoded primer, template solution and 0.5 μL Phusion® High-Fidelity DNA Polymerase (NEB, cat#M0530S). PCR was performed under the following thermocycler setting: a) 95 ℃ for 30 s, b) 95 ℃ for 10 s, c) 60.5 ℃ for 15 s, d) 72 ℃ for 30 s, e) repeat steps b) - d) for 25 times, f) 72 ℃ for 5 min and g) hold at 4 ℃.
